# Cellulosic ethanol production by consortia of *Scheffersomyces stipitis* and engineered *Zymomonas mobilis*

**DOI:** 10.1186/s13068-021-02069-8

**Published:** 2021-11-25

**Authors:** Lingling Sun, Bo Wu, Zengqin Zhang, Jing Yan, Panting Liu, Chao Song, Samina Shabbir, Qili Zhu, Shihui Yang, Nan Peng, Mingxiong He, Furong Tan

**Affiliations:** 1 Key Laboratory of Development and Application of Rural Renewable Energy, Ministry of Agriculture and Rural Affairs, Biogas Institute of Ministry of Agriculture and Rural Affairs, Chengdu, 610041 China; 2 Graduate School of Chinese Academy of Agricultural Sciences, Beijing, 100081 China; 3 Hubei Collaborative Innovation Center for Green Transformation of Bio-Resources, Environmental Microbial Technology Center of Hubei Province, Hubei Key Laboratory of Industrial Biotechnology, College of Life Sciences, Hubei University, Wuhan, 430062 China; 4 State Key Laboratory of Agricultural Microbiology, College of Life Science and Technology, Huazhong Agricultural University, Wuhan, 430070 China; 5 Chengdu National Agricultural Science and Technology Center, Chengdu, 610221 China

**Keywords:** *S. stipitis*, *Z. mobilis*, Carbon catabolite repression, Ethanol fermentation, Corn stover hydrolysate

## Abstract

**Background:**

As one of the clean and sustainable energies, lignocellulosic ethanol has achieved much attention around the world. The production of lignocellulosic ethanol does not compete with people for food, while the consumption of ethanol could contribute to the carbon dioxide emission reduction. However, the simultaneous transformation of glucose and xylose to ethanol is one of the key technologies for attaining cost-efficient lignocellulosic ethanol production at an industrial scale. Genetic modification of strains and constructing consortia were two approaches to resolve this issue. Compared with strain improvement, the synergistic interaction of consortia in metabolic pathways should be more useful than using each one separately.

**Results:**

In this study, the consortia consisting of suspended *Scheffersomyces stipitis* CICC1960 and *Zymomonas mobilis* 8b were cultivated to successfully depress carbon catabolite repression (CCR) in artificially simulated 80G40XRM. With this strategy, a 5.52% more xylose consumption and a 6.52% higher ethanol titer were achieved by the consortium, in which the inoculation ratio between *S. stipitis* and *Z. mobilis* was 1:3, compared with the *Z. mobilis* 8b mono-fermentation. Subsequently, one copy of the xylose metabolic genes was inserted into the *Z. mobilis* 8b genome to construct *Z. mobilis* FR2, leading to the xylose final-consumption amount and ethanol titer improvement by 15.36% and 6.81%, respectively. Finally, various corn stover hydrolysates with different sugar concentrations (glucose and xylose 60, 90, 120 g/L), were used to evaluate the fermentation performance of the consortium consisting of *S. stipitis* CICC1960 and *Z. mobilis* FR2. Fermentation results showed that a 1.56–4.59% higher ethanol titer was achieved by the consortium compared with the *Z. mobilis* FR2 mono-fermentation, and a 46.12–102.14% higher ethanol titer was observed in the consortium fermentation when compared with the *S. stipitis* CICC1960 mono-fermentation. Furthermore, qRT-PCR analysis of xylose/glucose transporter and other genes responsible for CCR explained the reason why the initial ratio inoculation of 1:3 in artificially simulated 80G40XRM had the best fermentation performance in the consortium.

**Conclusions:**

The fermentation strategy used in this study, i.e., using a genetically modified consortium, had a superior performance in ethanol production, as compared with the *S. stipitis* CICC1960 mono-fermentation and the *Z. mobilis* FR2 mono-fermentation alone. This result showed that this strategy has potential for future lignocellulosic ethanol production.

**Supplementary Information:**

The online version contains supplementary material available at 10.1186/s13068-021-02069-8.

## Background

Lignocellulosic biomass is produced by plant photosynthesis from solar energy and is the most abundant renewable feedstock in the world. The bio-conversion of lignocellulosic biomass into ethanol is viewed as one of the most promising ways to partially replace traditional fossil fuels, since the combustion of ethanol produces less particulate matter, carbon monoxide, and hydrocarbons than fossil fuels [1]. In addition, as bioethanol has a high octane number, mixing bioethanol with gasoline could improve the anti-detonating quality of transportation fuel.

Lignocellulosic biomass is primarily degraded into glucose and xylose after pretreatment and enzymatic hydrolysis. The simultaneous and efficient bioconversions of the two sugars into ethanol are the prerequisites for large-scale production of cellulosic ethanol. However, due to carbon catabolite repression (CCR), a considerable amount of wild microbes and engineered microbes having exogenous xylose–metabolic pathways, such as *Zymomonas mobilis*, *Escherichia coli*, *Saccharomyces cerevisiae* and *Bacillus amyloliquefaciens*, prefer to use glucose, and therefore, their xylose utilization generally lags behind glucose utilization [2–5]. This greatly hampers the large-scale application of cellulosic ethanol in industry.

Extensive studies have been attempted to relax CCR. For example, as all known xylose transporters are suppressed by glucose, many researchers have tried to engineer glucose-insensitive xylose transporters by evolutionary engineering, error-prone PCR, and site-directed mutagenesis. By this way, researchers have successfully built Gal2-N376F, CiGXS1 FIVFH497* and AN25-R4.18 [6–8]. In addition, adaptive evolution, computation simulation, and rational design have been used to find appropriate intracellular targets to alleviate CCR, such as the phosphoenolpyruvate transferase system (PTS), the cyclic AMP receptor protein (CRP), and the xylose operon regulatory protein [9, 10]. However, the complex nature of CCR makes it difficult to entirely reveal how CCR functions. Moreover, those above engineered strains developed cannot co-utilize glucose and xylose with high efficiency [9].

An alternative method is to build artificial consortia to co-ferment glucose and xylose. A common practice is to build a consortium consisting of a xylose-specific strain due to the deficiency in the PTS system and a wild strain that utilizes glucose because of CCR [9]. In this way, 20.82 g/L butanol with a yield of 0.35 g/g was produced from glucose and xylose using two *E. coli* strains. These results were comparable with butanol titers and yields produced in previous studies from glucose alone [11]. Another strategy is to use two wild species to ferment the glucose and xylose mixture. For example, when *Scheffersomyces stipitis* and *S*. *cerevisiae* were co-fermented, the xylose removal efficiency and ethanol production showed remarkable improvement than in their mono-fermentation [12]. In comparison with two-stage fermentation (glucose and xylose are consumed in a separate fashion), the consortium fermentation was advantageous in terms of assimilating glucose and xylose concomitantly and shortening fermentation time. Compared with strain improvement, the consortia might realize the exchange and transmission of substances, signals and energy between different cells, improve the adaptability and robustness of the population to complex environment. The synergistic interaction of their metabolic pathways also should be more useful than using each one separately. However, the challenge of consortia is selecting suitable strains which minimize the probable negative interactions.

*Zymomonas mobilis* is an excellent ethanol-producing species whose ethanol production efficiency can reach as high as 98%, which is higher than *S*. *cerevisiae* [13]. However, the wild *Z. mobilis* cannot utilize xylose unless it has been transformed by the exogenous xylose metabolic pathway, such as *Z. mobilis* 8b [14]. In addition, *S. stipitis* is recognized as one of the best microbes in nature in terms of its xylose assimilation ability, but it has a severe CCR phenomenon. In recent studies, the consortium of *S. stipitis* and *Z. mobilis* has been studied for the co-fermentation of glucose and xylose. However, these studies generally involved two-stage fermentation, or the total sugar concentrations in lignocellulosic hydrolysate medium were low, which is unrealistic in large-scale fermentation [15–18]. Even for simulated medium containing pure sugars, the xylose removal efficiency still requires improvement [19].

The present work focuses on investigating the potential of consortium fermentation consisting of *S. stipitis* and *Z. mobilis* in a simulated medium and corn stover hydrolysate, with a great emphasis on alleviating CCR, increasing the sugar removal efficiency, and increasing the bioethanol production. The corn stover was chosen since it is one of the most common annual agricultural wastes produced in China [20]. Transforming corn stover into industrial products, such as ethanol, not only realizes the resource utilization of this waste, but also contributes to the progress of global warming mitigation.

## Results and discussion

### Co-culture of *S. stipitis *CICC1960 and *Z. mobilis* 8b in 80G40XRM

Artificially simulated 80G40XRM (80 g/L glucose + 40 g/L xylose) was first used to explore the appropriate mode of this consortium fermentation with the aim of improving the glucose-and-xylose co-utilization efficiency. The ratio between glucose and xylose in this medium was 2:1, aiming to simulate the real ratio in lignocellulosic hydrolysates [21].

**Fig. 1:**
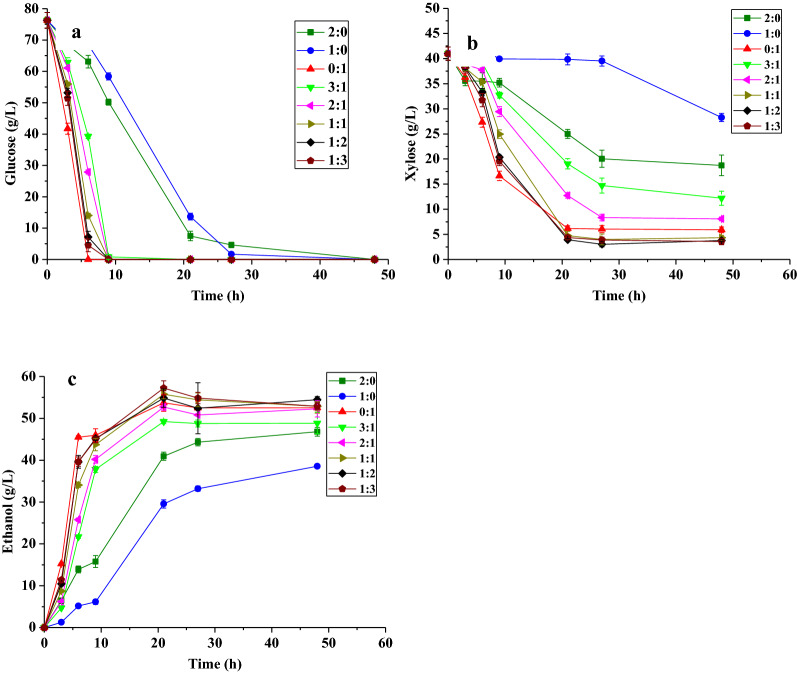
Fermentation profiles of consortia consisting of *S. stipitis* CICC1960 and *Z. mobilis* 8b in 80G40XRM with “high density” inoculums of fermentations. **a** Glucose assimilation profiles. **b** Xylose assimilation profiles. **c** Ethanol production profiles. Data are mean ± standard error from four replicates

In a previous study [22], when *S. stiptis* was pre-cultured in the medium with glucose as the sole carbon source (glucose medium), its xylose–metabolic gene expression, such as D-xylose reductase and xylitol dehydrogenase expression, was inhibited. Hence, when *S. stiptis* was inoculated in the xylose medium (xylose was the sole carbon source in the fermentation medium) later, its xylose–metabolic genes needed to be synthesized from scratch. Therefore, *S. stiptis* exhibited an apparent lag in fermentation in the xylose medium. In contrast, when *S. stiptis* was precultured in the xylose medium, the two xylose–metabolic genes were fully expressed and the aforementioned problem of lag in the xylose fermentation was alleviated, especially if *S. stiptis* was inoculated with high initial density, such as OD_620_ = 40. To this end, we decided to preculture *S. stiptis* CICC1960 in YP120X (120 g/L xylose) and then inoculate it to the 80G40XRM (fermentation medium) with a “high density” and a “low amount” of inoculum fermentations, hoping to alleviate the CCR phenomenon in *S. stiptis*. However, as shown in Fig. 1 (with “high density” inoculums) and Additional file 1: Fig. S1 (with “low amount” inoculums), *S. stipitis* CICC1960 still could not simultaneously assimilate glucose and xylose. This might have been a result of the glucose repression on xylose transportation into the cells [6], or the inoculum size used in this study (the initial OD_600_ in fermentation was about 1.8) was not high enough. For economic reasons, the inoculum size was not further increased in this study.

For *Z. mobilis* 8b, 80G40XRM was used as its seed culture medium, since *Z. mobilis* 8b cannot grow well in xylose medium (data not shown). The results of the *Z. mobilis* 8b mono-fermentation are shown in Fig. 1 and Additional file 1: Table S1 and Fig. S1. During fermentation with “high density” of inoculum, *Z. mobilis* 8b showed a strong ability to utilize glucose and xylose simultaneously with ethanol productivity reaching 5.10 g/L/h. More specially, the CCR in *Z. mobilis* 8b was largely alleviated, which may be attributed to the high inoculum size used in this study (the initial OD_600_ was approximately 1.8). In 2004, Ali Mohagheghi et al. found that *Z. mobilis* 8b (the initial inoculum density was 0.2 at 600 nm) exhibited an apparent lag of xylose utilization, while glucose could be assimilated immediately [23]. Since the xylose–metabolic genes were inserted to the genome of *Z. mobilis* 8b, their expression was generally low in individual cells. When *Z. mobilis* 8b were inoculated with high density, activities of those enzymes encoding xylose–metabolic genes would be higher than the ones under a low initial density inoculation, and this may contribute to the alleviation of CCR in this study [14]. Glucose was completely removed within 6 h by *Z. mobilis* 8b, while xylose still remained at 6.16 g/L at 21 h. The ethanol yield of the *Z. mobilis* 8b mono-fermentation was 0.48 g/g (the theoretical ethanol yield is 0.51 g/g sugars).

When *S. stipitis* CICC1960 and *Z. mobilis* 8b were co-cultured together with different inoculation ratios (3:1–1:3) with “high density” of inoculum, the glucose consumption profiles of the consortia did not differ considerably from the *Z. mobilis* 8b mono-fermentation (Fig. 1a). Glucose was completely removed within 9 h by the consortia. On top of this, no apparent CCR was shown since xylose was rapidly assimilated at the very early stage (Fig. 1b). Although xylose consumption rates by the consortia were lower than that in the *Z. mobilis* 8b mono-fermentation. It was shown that, in general, the higher the ratio of *Z. mobilis* 8b applied, the higher the rates of xylose assimilation achieved. Specially, when *S. stipitis* CICC1960:*Z. mobilis* 8b = 3:1 (initial inoculum size proportion), the xylose assimilation rate was 0.92 g/L/h; when *S. stipitis* CICC1960: *Z. mobilis* 8b = 1:3, the xylose assimilation rate improved to 2.38 g/L/h. At 21 h, the consortia fermentation reached the endpoint of fermentation. Interestingly, when *S. stipitis* CICC1960:*Z. mobilis* 8b = 1:3, the xylose consumption reached 36.73 g/L, which was significantly (P < 0.01) higher than that in the *S. stipitis* CICC1960 mono-fermentation and that in the *Z. mobilis* 8b mono-fermentation (Additional file 1: Table S1). Correspondingly, the ethanol titer of this consortium reached 57.21 g/L, which was 48.37% higher than that of the *S. stipitis* CICC1960 mono-fermentation and 6.52% higher than that of the *Z. mobilis* 8b mono-fermentation. These results (*S. stipitis* CICC1960:*Z. mobilis* 8b = 1:3) were comparable with or better than other consortia fermentations listed in Table 1, in terms of xylose removal efficiency, ethanol yield, and ethanol productivity. To explain the best results observed were not due to the inoculum size of *Z. mobilis*, but to the presence of *S. stipitis*, the results of “*S. stipites*: *Z. mobilis* = 2:0” were compared with “*S. stipitis*: *Z. mobilis* = 1:1”. The results (Fig. 1) showed that the xylose utilization and ethanol yield of higher initial inoculation amount of *S. stipitis* (2:0) were better than that of 1:0 inoculation amount; however, it was still not better than that of the consortium (*S. stipitis*: *Z. mobilis* = 1:1) and other consortia.

**Table 1:** Comparison of various consortia fermentation profiles in simulated medium

Fermentation mode^a^	Initial sugar concentration (g/L)	Xylose removal efficiency (%)	Ethanol yield (g/g)	Ethanol productivity (g/L/h)	References
Two-stage fermentation					
Suspended *Z. mobilis* + suspended *S. stipitis*	80 g/L glucose + 40 g/L xylose	62.5	–	1.56	[15]
Suspended *S. stipitis* + suspended *Z. mobilis*^b^	60 g/L xylose + 100 g/L glucose	–	0.474	1.416	[16]
Suspended *Z. mobilis* + suspended *S. stipitis*	80 g/L glucose + 40 g/L xylose	0.67	0.36	0.41	[19]
One-stage fermentation					
Suspended *S. cerevisiae* + suspended *S. stipitis*	75 g/L glucose + 30 g/L xylose	79.6	0.4	1.26	[41]
Suspended *S. cerevisiae* + suspended *S. stipitis*	20 g/L glucose + 10 g/L xylose	–	0.416	0.608	[42]
Immobilized *Z. mobilis* + immobilized *S. stipitis*^c^	80 g/L glucose + 40 g/L xylose	72.5	0.37	0.87	[19]
Suspended *Z. mobilis* + suspended *S. stipitis*^d^	80 g/L glucose + 40 g/L xylose	84.95	0.50	4.99	This study

^a^Two-stage fermentation means the two species were inoculated into the medium sequentially, while one-stage fermentation means the two species were inoculated into the medium simultaneously

^b^Xylose was first depleted by *S. stipitis*. Then, glucose medium and *Z. mobilis* were added to the same system to initiate the glucose fermentation

^c^*Z. mobilis*and *S. stipitis*were immobilized separately

^d^ *S. stipitis* CICC1960:*Z. mobilis* 8b = 1:3

–, not available

Due to the outstanding fermentation ability of this consortium (*S. stipitis* CICC1960:*Z. mobilis* 8b = 1:3), the ratio between *S. stipitis* and *Z. mobilis* of 1:3 was later employed in the fermentation of corn stover hydrolysate.

### Genetic engineering of *Z. mobilis* 8b

In *Z. mobilis*, ZMO0256 encoding D-lactate dehydrogenase is involved in the production of lactate as a byproduct; ZMO0689 encoding glucose–fructose oxidoreductase participates in xylitol and sorbitol production [24]. It was demonstrated that disruption of ZMO0689 could improve xylose fermentation performance of *Z. mobilis* [25]. To introduce one copy of xylose metabolic genes (*xylA*, *xylB*, *tktA*, *talB*) into the *Z. mobilis* 8b genome and improve its xylose assimilation performance, the engineered strains *Z. mobilis* FR1 (ZMO0256::P_*pdc*_-*talB*-*tktA*) and *Z. mobilis* FR2 (ZMO0256::P_*pdc*_-*talB*-*tktA*; ZMO0689::P_*pdc*_-*xylA*-*xylB*) were sequentially constructed.

**Fig. 2:**
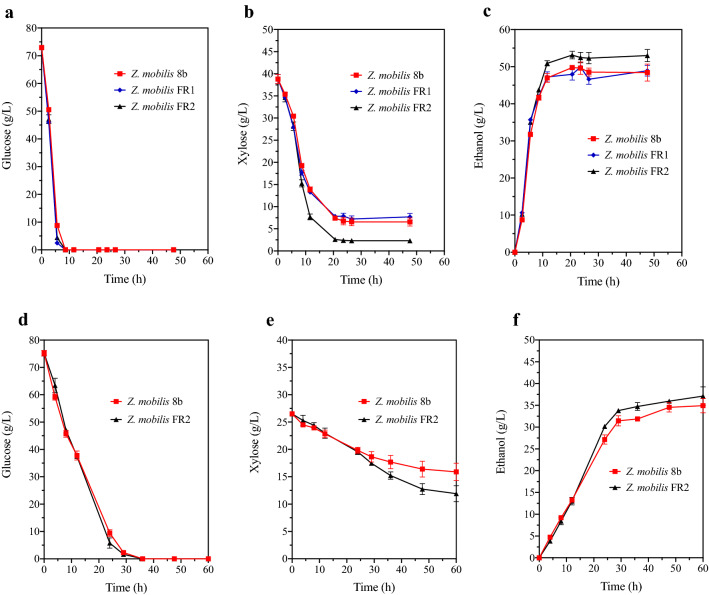
Fermentation profiles of *Z. mobilis* 8b, *Z. mobilis* FR1, and *Z. mobilis* FR2 in 80G40XRM and in corn stover hydrolysate. **a**, **b**, **c** Glucose**,** xylose, and ethanol production, respectively, in 80G40XRM; **d**, **e**, **f** Glucose, xylose and ethanol production in corn stover hydrolysate. The total concentration of glucose and xylose in the hydrolysate was 120 g/L before autoclave sterilization. Data are mean ± standard error from four replicates

The fermentation performances of *Z. mobilis* FR1 and *Z. mobilis* FR2 were evaluated in 80G40XRM and were compared with their parental stain *Z. mobilis* 8b. As shown in Fig. 2a, the three strains did not differ in their glucose assimilation profiles, and glucose was depleted within 8.5 h. For the xylose consumption and ethanol production profiles (Fig. 2b, c), *Z. mobilis* FR1 did not show much difference with *Z. mobilis* 8b. However, *Z. mobilis* FR2 accelerated its xylose assimilation rate in the mid-to-late fermentation period (Fig. 2b): the xylose consumption and ethanol production achieved by *Z. mobilis* FR2 were increased by 15.36% and 6.81%, respectively, at 20.5 h as compared with *Z. mobilis* 8b (Additional file 1: Table S2). Besides, the ethanol productivity of *Z. mobilis* FR2 was 5.08 g/L/h, which was significantly (P < 0.01) higher than *Z. mobilis* 8b (4.84 g/L/h). The higher xylose-to-ethanol transforming rate in *Z. mobilis* FR2 fermentation could be attributed to the extra introduction of four xylose metabolic genes (*xylA*, *xylB*, *tktA*, *talB*). As the expression of the four genes are all controlled under the constitutive promoter *pdc*, the expression levels of their protein products (xylose isomerase, xylulokinase, transketolase, transaldolase) in *Z. mobilis* FR2 are higher than its parental strain *Z. mobilis* 8b. Therefore, xylose was transformed into fructose-6-P and flyceraldehyde-3-P and then entered the Entner–Doudoroff pathway in a greater speed (Additional file 1: Fig. S2), and finally contributes to the improvement of ethanol productivity in *Z. mobilis* FR2 fermentation. The ethanol yield of *Z. mobilis* FR2 was 95.47%, which was comparable with A3 (96.6%) and AD50 (96%), the two best strains developed so far by adaptive laboratory evolution [24, 26]. However, as shown in Fig. 2a, b, though CCR was alleviated in *Z. mobilis* FR2 fermentation, its xylose utilization rate was still lower than its glucose utilization rate, and there was still a slight amount of xylose that remained (approximately 2.5 g/L) at the endpoint. These results agreed with other *Z. mobilis* strains, including C25, 39,676/pZB4L [27], ZM4/Ac^R^ (pZB5, pJX1) [28], A3 [24], and AD50 [26], yet no exact reason of the incomplete xylose utilization has been identified thus far. In *Z. mobilis*, xylose is transported through a glucose facilitated diffusion protein [26], which is a native glucose transporter and has low affinity to xylose. This low affinity xylose transport might be the burden behind the above-mentioned problems. Further investigation into this field would greatly promote the commercialization of cellulosic ethanol.

### Effect of oxygen on *Z. mobilis* FR2 fermentation

**Fig. 3:**
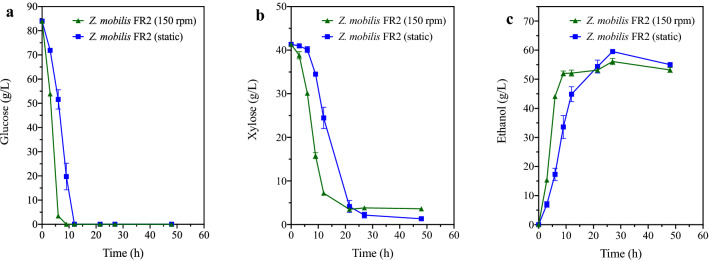
Fermentation profiles of *Z. mobilis* FR2 in 80G40XRM with different rotation speeds. **a** Glucose assimilation profiles. **b** Xylose assimilation profiles. **c** Ethanol production profiles. Data are mean ± standard error from four replicates

In the above experiments, as *S. stipitis* could not grow under static cultivation, 150 rpm was applied in the *S. stipitis* mono-fermentation, the *Z. mobilis* mono-fermentation, and the consortia fermentation that consisted of the two species. However, as *Z. mobilis* is a facultative anaerobe, it can ferment under both static and aerobic conditions. Therefore, a further study was conducted to check whether there was any difference in *Z. mobilis* FR2 fermentation profiles under static and agitated (150 rpm) conditions in 80G40XRM. As shown in Fig. 3, oxygen significantly boosted *Z. mobilis* FR2’s glucose (P < 0.05) and xylose (P < 0.01) consumption rates and improved ethanol productivity by 54.51% (P < 0.01). However, at fermentation endpoint (27 h), the static fermentation of *Z. mobilis* FR2 showed an increase in xylose consumption and ethanol production by 1.65 g/L and 3.45 g/L, respectively, compared with that in agitated fermentation. To achieve high ethanol productivity, ethanol fermentation under 150 rpm was kept in this study.

Since the theoretical ethanol yield is 0.51 g/g xylose, the 1.65 g/L more xylose consumed by *Z. mobilis* FR2 in static condition, in theory, should be transformed into 0.84 g/L more ethanol. However, the real difference in ethanol titers between the static fermentation and agitated fermentation was as high as 3.45 g/L. This suggests oxygen had a negative effect on ethanol production by *Z. mobilis* FR2. In 1990, Tanaka et al. found that *Z. mobilis* produced a little more acetaldehyde (0.28–4.49 g/L) when oxygen was supplied [29]. Acetaldehyde was primarily produced by NADH dehydrogenase, one of the key components in the *Z. mobilis* respiratory chain. This enzyme has the same cofactor (NADH) as ethanol dehydrogenase. During aerobic fermentation, the activity of NADH dehydrogenase was higher than that of ethanol dehydrogenase. Therefore, a large amount of NADH was used to reduce the dissolved oxygen concentration in the medium. Due to the lack of sufficient NADH, the transformation from acetaldehyde to ethanol by ethanol dehydrogenase was inhibited, and thus negatively affected the ethanol production of *Z. mobilis* under agitated cultivation [30]. One evidence for this hypothesis is that for *Z. mobilis* mutant strains, whose NADH dehydrogenase is defective, this negative effect of oxygen on *Z. mobilis* ethanol fermentation could be alleviated [31–33].

### Assimilation of corn stover hydrolysates by consortium composed of *S. stipitis* CICC1960 and *Z. mobilis* FR2

First, it was evaluated whether *Z. mobilis* FR2 could outcompete *Z. mobilis* 8b in corn stover hydrolysate fermentation. As shown in Fig. 2d a difference was not observed regarding glucose assimilation ability of the two strains: glucose assimilation rates were 2.54 g/L/h and 2.51 g/L/h for *Z. mobilis* FR2 and *Z. mobilis* 8b, respectively, and glucose was depleted within 30 h. As for xylose assimilation (Fig. 2e), though the two strains did not exhibit a difference during the first 24 h, the assimilation rate of *Z. mobilis* 8b gradually decreased, while the rate for *Z. mobilis* FR2 remained stable. At 60 h, *Z. mobilis* FR2 assimilated 14.60 g/L xylose, which was significantly higher (P < 0.05) than that of *Z. mobilis* 8b (10.60 g/L). In addition, *Z. mobilis* FR2 produced 37.13 g/L ethanol in 120 g/L corn stover hydrolysate (concentration here refers to the total amount of glucose and xylose in corn stover hydrolysate before sterilization), while *Z. mobilis* 8b produced 34.93 g/L ethanol (Fig. 2f). These results agreed with the fermentation results in 80G40XRM (Fig. 2a, b, c). Due to the better xylose assimilation ability of *Z. mobilis* FR2, *Z. mobilis* FR2 was used to replace *Z. mobilis* 8b in the next consortium fermentation with *S. stipitis*CICC1960 in corn stover hydrolysates.

**Fig. 4:**
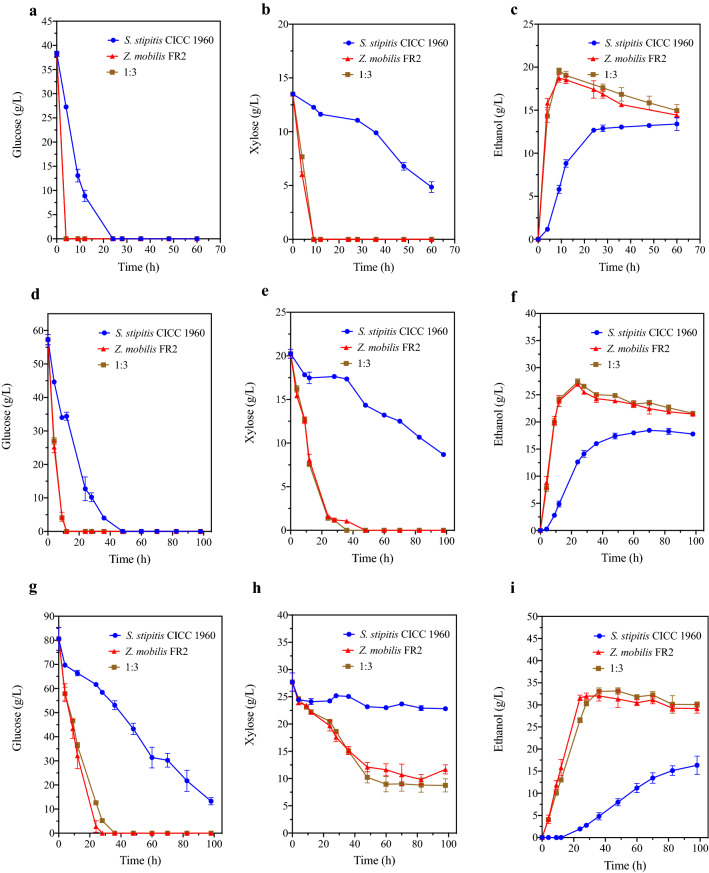
Consortium fermentation profiles in variousconcentrations of corn stover hydrolysates. **a**, **b**, **c** Glucose, xylose and ethanol fermentation profiles in 60 g/L of corn stover hydrolysate. **d**, **e**, **f** Glucose, xylose and ethanol fermentation profiles in 90 g/L of corn stover hydrolysate. **g**, **h**, **i** Glucose, xylose and ethanol fermentation profiles in 120 g/L of corn stover hydrolysate.The concentration of corn stover hydrolysates represents the total amount of glucose and xylose in the hydrolysates before autoclave sterilization. For consortium fermentation, the inoculation ratio between *S. stipitis* CICC1960 and *Z. mobilis* FR2 was 1:3. Data are mean ± standard error from three replicates

As shown in Fig. 4, while consortium fermentation (*S. stipitis* CICC1960:*Z. mobilis* FR2 = 1:3) and *Z. mobilis* FR2 mono-fermentation did not show any difference in glucose and xylose consumption rates and amounts in the 60 and 90 g/L corn stover hydrolysates fermentation, the consortium produced slightly more ethanol (~ 0.86 g/L) than the *Z. mobilis* FR2 mono-fermentation (Table 2). In addition, the consortium fermentation in the two cases was better than the *S. stipitis* CICC1960 mono-fermentation in terms of glucose assimilation, xylose assimilation, and ethanol production rates and quantities, and did not exhibit strong CCR which was evident in the *S. stipitis* CICC1960 mono-fermentation (Fig. 4). For the 120 g/L corn stover hydrolysate fermentation, the glucose assimilation rate of the consortium (2.83 g/L/h) was slightly slower than *Z. mobilis* FR2 (3.24 g/L/h), while xylose assimilation rates were nearly the same prior to 36 h. However, the consortium finally produced 33.05 g/L ethanol at endpoint, which was 1.02 g/L higher than the *Z. mobilis* FR2 mono-fermentation and 16.7 g/L higher than the *S. stipitis* CICC1960 mono-fermentation (Table 2).

**Table 2:** Fermentation profiles in the corn stover hydrolysate

Corn stover hydrolysate concentration (g/L)^a^	*S. stipitis* CICC1960:*Z. mobilis* FR2	Glucose consumed (g/L)^b^	Xylose consumed (g/L)^b^	Ethanol^b^		
				Titer (g/L)	Yield (g/g)	Productivity (g/L/h)
60	1:0	38.27 ± 0.53	8.64 ± 0.49	13.40 ± 0.75	0.29 ± 0.01	0.73 ± 0.04
	0:1	38.27 ± 0.53	13.50 ± 0.00	18.72 ± 0.46	0.36 ± 0.01	2.08 ± 0.05
	1:3	38.27 ± 0.53	13.50 ± 0.00	19.58 ± 0.32	0.38 ± 0.01	2.18 ± 0.04
90	1:0	57.30 ± 1.55	7.74 ± 0.25	18.45 ± 0.35	0.28 ± 0.01	0.53 ± 0.01
	0:1	57.30 ± 1.55	18.62 ± 0.15	26.96 ± 0.24	0.36 ± 0.00	1.99 ± 0.08
	1:3	57.30 ± 1.55	18.87 ± 0.04	27.38 ± 0.58	0.36 ± 0.01	2.01 ± 0.05
120	1:0	67.35 ± 1.52	4.88 ± 0.19	16.35 ± 2.06	0.23 ± 0.03	0.19 ± 0.02
	0:1	80.61 ± 4.72	12.56 ± 0.74	32.03 ± 1.14	0.34 ± 0.01	1.32 ± 0.03
	1:3	80.61 ± 4.72	12.69 ± 0.60	33.05 ± 0.79	0.35 ± 0.01	1.11 ± 0.02

Data are mean ± standard error from three replicates

^a^The corn stover hydrolysate concentration refers to the total concentrations of glucose and xylose in the hydrolysate before autoclave sterilization

^b^All data were calculated based on the real sugar concentrations

In 2014, Lalit K. Singh et al. separated kans grass hydrolysate into a xylose-rich portion and a glucose-rich portion through organic solvent extraction and then used *S. stipitis* and *Z. mobilis* to ferment each sugar (54 g/L xylose and 100 g/L glucose) sequentially. The ethanol productivity in their study was 0.723 g/L/h, which was lower than that in our study (1.11–2.18 g/L/h) [16]. This was because the two-stage fermentation Lalit K. Singh et al. employed led to an increase in the fermentation time. In 2020, Ferdian Wirawan et al. used immobilized *Z. mobilis* and *S. stipitis* to sequentially ferment 50 g/L sugarcane bagasse [17]. Though the productivity in their study was high (1.868 g/L/h), the actual sugar concentration in the hydrolysate was only 11 g/L glucose, 4 g/L xylose, and 4 g/L cellobiose, which is impractical in real applications. These comparisons demonstrated that the consortium fermentation mode utilized in this study has an edge in ethanol production compared with other existed modes of *S. stipitis* and *Z. mobilis* co-fermentation.

We note that the ethanol titer and productivity values in the 120 g/L corn stover hydrolysate were lower than the values in 80G40XRM (Table 2, Additional file 1: Tables S1 and S2). This was because many of the inhibitors, such as phenols, were presented in the corn stover hydrolysate [34]. These inhibitors negatively affected the microbial fitness in the lignocellulosic hydrolysate and thus reduced the ethanol yield and productivity. Although it has been shown that the immobilization of microbes could alleviate the negative effect to some degree, the exact mechanisms are not clear [35].

Duong Thi Thuy Nguyen et al. found that the presence of living *Z. mobilis* cells negatively affected the xylose assimilation performance of *S. stipitis*, suggesting that there might be an amensalism relationship between the two species [19, 36]. Similarly, in our study, when the initial inoculum OD_600_ was controlled to 0.1 in the 80G40XRM fermentation, no improvement was observed in the consortium fermentation profiles compared with the *Z. mobilis* mono-fermentation profiles (data not shown). In contrast, when a high inoculum size (an initial OD_600_ of approximately 1.8) was used, the consortium of *S. stipitis* and *Z. mobilis* assimilated more xylose and produced more ethanol in both the 80G40XRM and 120 g/L corn stover hydrolysate. This implied a commensalism or cooperation relationship between the two species during fermentation [36]. Perhaps this positive relationship could only be exhibited under specific conditions, such as under a high cell density.

Exploring the interactions between *S. stipitis* and *Z. mobilis* under various cell densities, as well as the ethanol fermentation performance in corn stover hydrolysate by co-immobilized cells of the two species, are our future research goals. Indeed, a deep understanding of the interactions between *S. stipitis* and *Z. mobilis* and the mechanisms behind the protective role of co-immobilization on cells will pave the way to further enhance the consortium fermentation performance while lowering the inoculum size, and will help promoting the industrial progression of cellulosic ethanol production.

### Analysis of gene expression by qRT-PCR

**Fig. 5:**
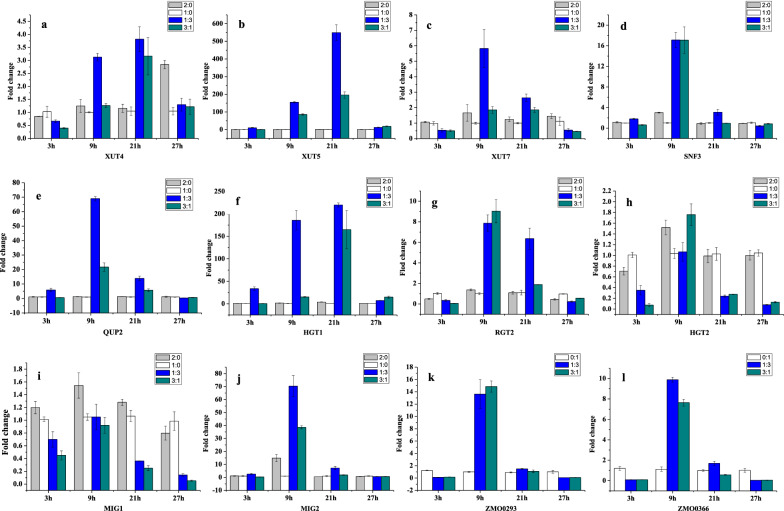
Fold changes in xylose/glucose transporters and CCR gene expression levels of consortia and control mono-fermentation. **a**, **b**, **c** Xylose transporter in *S. stipitis*; **d**, **e**, **f**, **g**, **h** Glucose transporter in *S. stipitis*; **i**, **j** Carbon catabolite repressor (CCR) in *S. stipitis*; **k**, **l** Glucose/xylose transporter in *Z. mobilis*.

As for the outstanding fermentation ability of the inoculum ratio 1:3 between *S. stiptis* and *Z. mobilis* with “high density”, genes of xylose and glucose transporters in two strains and carbon catabolite repressor (CCR) in *S. stipitis* were chosen to qRT-PCR analysis. Samples were taken at 3, 9, 21 and 27 h, respectively, and the gene expressions in the consortia were compared with that of corresponding mono-fermentation at the same time. As shown in Fig. 5, at the early stage of fermentation (3 h), genes related to xylose and glucose transporters in *S. stipitis* (*XUT4, XUT5, XUT7, SNF3, QUP2, RGT2, HGT1, HGT2*) and *Z. mobilis* (*ZMO 0366, ZMO 0293*) had no significant differences between the consortium (1:3) and mono-fermentation. However, these genes in the consortium (1:3) were up-regulated to some different extent compared with that in mono-fermentation at 9 h and 21 h. For example, *XUT5* and *XUT7* related xylose transporter of *S. stipitis* in the consortium (1:3) had a 155.1- and 5.8-fold enhanced expression at 9 h, respectively, compared to the mono-fermentation of *S. stipitis*, even 549.5-fold for *XUT5* at 21 h. Among glucose transporter related genes including *SNF3, QUP2, RGT2* and *HGT1* of *S. stipitis* in the consortium (1:3) were up-regulated about 17.1-, 68.9-, 7.9-, and 185.6-fold at 9 h, respectively, compared to *S. stipitis* mono-fermentation. At the same time, the gene *ZMO0366* (glucose facilitated diffusion protein) and *ZMO0293* (sugar porter family MFS transporter) came from *Z. mobilis* also had about 9.9-, 13.6-fold up-regulation at 9 h in the consortium (1:3), respectively. These results above were in consistent with the results of fermentation ability, which could explain why the utilizations of xylose and glucose in the consortium (1:3) were better than that of *S. stipitis* mono-fermentation and *Z. mobilis* mono-fermentation (Fig. 1). The higher the expression of xylose or glucose transporter, the more xylose or glucose could be transported for utilization. In addition, the gene *ZMO 0366* and *ZMO 0293* also had about 7.9-, 14.8-fold up-regulation at 9 h in the consortium of 3:1, respectively, which were comparable to the consortium of 1:3. The finding showed that the up-regulation of genes related xylose and glucose transporters came from *S. stipitis* played an important role in the consortium of 1:3. It has been reported that Mig2 was identified as a repressor that collaborates with Mig1 to cause glucose-induced repression of *SUC2* gene [37]. Mig2 could be also involved in the cross talk between the nucleus and the mitochondria through Ups1 to regulate mitochondrial morphology in a glucose dependent manner [37, 38]. Interesting, the expression of *Mig1* gene came from *S. stipitis* was down-regulated at 9 h and 21 h in the consortium compared to *S. stipitis* mono-fermentation, while *Mig2* were up-regulated. Therefore, it could be speculated that the down-regulation of Mig1 could contribute to reducing CCR, and Mig2 might play a more important role compared to Mig1 in the consortium. From Fig. 5, we also found that the increase of initial inoculation of *S. stipitis* in mono-fermentation didn’t cause changes in the expression levels of xylose and glucose transporters and CCR genes, which was consistent with the fermentation results, that is, the increase of *S. stipitis* initial inoculation didn’t improve the fermentation performance of mono-fermentation. Gao et al. have reported that the initial ratio can influence the structure and, more importantly, the function and the bacterial interaction of a community [39]. Our results suggested that there might be an interaction between *S. stipitis* and *Z. mobilis* and the initial ratio of 1:3 is able to regulate the expression of genes of xylose and glucose transporters in two strains to better utilize glucose and xylose simultaneously.

## Conclusions

This study focused on evaluating a fermentation method to efficiently transform glucose and xylose to ethanol via artificial consortia composed of suspended *S. stipitis* and suspended *Z. mobilis*. By fermentation process optimization and genetic engineering, the consortium built here exhibited enhanced xylose assimilation ability and ethanol production performance in both 80G40XRM and corn stover hydrolysates, and did not experience evident CCR phenomenon. Hence, this study proved that *S. stipitis* and *Z. mobilis* could be co-cultured together in suspension in cellulosic ethanol production and provided a novel strategy for further application.

## Materials and methods

### Strains, plasmids and primers

*S. stipitis* CICC1960 was purchased from the China Center of Industrial Culture Collection. *Z. mobilis* 8b was kindly given by Shihui Yang, Hubei University, and was used as the starting strain for further strains constructions. Plasmid Pmini was kindly given by Nan Peng, Huazhong Agricultural University. All primers were synthesized by Tsingke Biotechnology Co., Ltd. (Chengdu, China) and purified via polyacrylamide gel electrophoresis.

All strains, plasmids and sgRNAs are listed in Additional file 1: Table S3. Primers are listed in Additional file 1: Table S4.

### Genetic engineering and strain development

The CRISPR-Cas system was used to engineer *Z. mobilis* FR1 and *Z. mobilis* FR2 in this study. Specifically, the plasmids Pmini-P_*pdc*_-*talB*-*tktA* and Pmini-P_*pdc*_-*xylA*-*xylB* were constructed using Gibson Assembly® Protocol (New England BioLabs, Ipswich, US) and were transformed into *E. coli* trans110 (TransGen Biotech, Beijing, China). After verification of the plasmid sequences by sequencing, the two plasmids were transformed into *Z. mobilis* 8b sequentially to construct *Z. mobilis* FR1 (ZMO0256::P_*pdc*_-*talB*-*tktA*) and *Z. mobilis* FR2 (ZMO0256::P_*pdc*_-*talB*-*tktA*; ZMO0689::P_*pdc*_-*xylA*-*xylB*). ZMO0256up500, *pdc* promoter, *talB*, *tktA*, ZMO0256down500, ZMO0689up540, *xylA*, *xylB*, and ZMO0689down500 were all amplified from *Z. mobilis* 8b, using the corresponding primers listed in Additional file 1: Table S4. Only the engineered strains confirmed by both gel electrophoresis and gene sequencing were used in the experiments.

### Preparation of the corn stover hydrolysates

#### Alkaline pretreatment [40]

Corn stover was collected from Jianyang, Sichuan, China. After air-drying, milling and passing through a sieve with an aperture size of 380 μm, the corn stover was mixed with 1.34% (w/v) NaOH solution (The solid loading was 10% (w/v)), loaded into reaction kettles, and transferred into a drying oven sequentially. The treatment parameters were 140 °C and 6 h.

The pretreated corn stover was washed intensively with water or diluted HNO_3_ until the pH of the washing water turned neutral. The washed corn stover was oven dried and stored in sealed bags at room temperature.

#### Enzymatic hydrolysis

Pretreated corn stover (10 g) was mixed with 100 mL citric acid buffer (8.823 g/L tri-sodium citrate dihydrate, 3.843 g/L citric acid, pH 4.8) and 5 mL cellulase (Sigma-Aldrich, Saint Louis, US). Afterwards, the mixture was placed in an incubator at 50 °C and 150 rpm for 72 h. When the enzymatic hydrolysis was finished, the hydrolysate was centrifuged twice at 3000 g for 25 min each time. The hydrolysate was then centrifuged again at 10,000 g for 5 min for the further removal of the remaining solids. Sugars in the lignocellulosic hydrolysate were concentrated using a rotary evaporator to attain 60 g/L, 90 g/L, and 120 g/L hydrolysates with regard to the concentrations of total glucose and xylose. Subsequently, 10 g/L yeast extract, 2 g/L KH_2_PO_4_, 1 g/L (NH_4_)_2_SO_4_, and 2 g/L MgSO_4_·7H_2_O were added to the hydrolysates before adjusting the pH of the hydrolysates to 5.6 by 5 M NaOH.

### Growth and fermentation conditions

#### Preparation of the seed cultures of *S. Stipitis* and *Z. mobilis*

*Scheffersomyces stipitis* was streaked in a YPD plate (20 g/L glucose, 10 g/L yeast extract, 20 g/L peptone, and 15 g/L agar) and cultured at 30 °C for approximately 1 day. A single colony was transferred from the YPD plate into 5 mL of YP120X medium (120 g/L xylose, 10 g/L yeast extract, and 20 g/L peptone) and cultivated at 30 °C and 150 rpm for 19–36 h. Afterwards, the entire pre-seed culture was inoculated into a 100 mL of fresh YP120X medium and cultivated at 30 °C and 150 rpm for 22 h. At this point, the OD_600_ value of the seed culture was approximately 1.8.

Similarly, *Z. mobilis* was streaked in a RM plate (20 g/L glucose, 10 g/L yeast extract, 2 g/L KH_2_PO_4_, 1 g/L (NH_4_)_2_SO_4_, 2 g/L MgSO_4_·7H_2_O, and 15 g/L agar) and cultivated at 30 °C for 2 days. A single colony was transferred from the RM plate into 5 mL of 80G40XRM (80 g/L glucose, 40 g/L xylose, 10 g/L yeast extract, 2 g/L KH_2_PO_4_, 1 g/L (NH_4_)_2_SO_4_, and 2 g/L MgSO_4_·7H_2_O) and cultivated statically at 30 °C for 19–36 h. The entire culture was then inoculated into 100 mL of the fresh 80G40XRM and cultivated statically at 30 °C for 22 h. The OD_600_ value of the seed culture was approximately 1.8 at this point.

#### Fermentation conditions

For the “high density” inoculation fermentation, the seed cultures of *S. stipitis* and *Z. mobilis* were centrifuged at 4 °C with 4000 rpm for 5 min, washed once with sterile ddH_2_O, and suspended with 1/25 (v/v) new fermentation medium, respectively (for 80G40XRM fermentation, 80G40XRM was used to suspend cells, while sterile ddH_2_O was used for corn stover hydrolysates fermentation). The concentrated cells were then inoculated into the corresponding fermentation medium with different ratios of *S. stipitis* and *Z. mobilis*. The total inoculum size was 100% (v/v, the ratio of seed culture volume/fermentation culture volume). For the “low amount” inoculation fermentation, after centrifuging and washing, the seed cultures of *S. stipitis* and *Z. mobilis* were added proper 80G40XRM to suspend cells to make OD_600_ value about 1.0.The total fermentation volume was 50 mL for 80G40XRM fermentation and 10 mL for corn stover hydrolysates fermentation. The fermentation conditions were 30 °C and 150 rpm, unless there was further indication in the portion for exploring the effect of oxygen on *Z. mobilis* fermentation (as seen in Section “Effect of oxygen on *Z. mobilis* FR2 fermentation”).

Four replicates were performed for the 80G40XRM fermentation, while three replicates were performed for the corn stover hydrolysates fermentation.

### Analytical methods

#### Determination of glucose, xylose and ethanol concentrations during fermentation

The glucose, xylose, and ethanol titers in the fermentation samples were analyzed by an Agilent 1200 Series HPLC system (Agilent Technologies, Santa Clara, US) equipped with a Bio-Rad HPX-87H column (Bio-Rad Laboratories, Richmond, US). The mobile phase was 5 mM H_2_SO_4_. The operating parameters were 20 μL injection volume, 0.6 mL/min rate, and 35 °C. The ethanol productivity and yield were calculated using the following formulas.

- (1) Ethanol productivity = Ethanol titer/fermentation time.
- (2) Ethanol yield = Ethanol titer/glucose and xylose consumed.

The theoretical ethanol yield is 0.51 g/g sugars consumed.

#### Quantitative real-time reverse transcription PCR

Total cellular RNA was extracted from cells grown for 3 h, 9 h, 21 h and 24 h using Trizol reagent (Invitrogen). Reverse transcription step was carried out using Goldenstar RT6 cDNA Synthesis Kit Ver 2 (TsingKe, Chengdu, China) with random primer mix following the manufacturer’s manual. Quantitative real-time reverse transcription PCR was performed in the Bio-Rad iQ5 real-time PCR detection system with SuperReal PreMix (SYBR Green) (TIANGEN, Beijing, China). All optimized primers are shown in Table S4 and were designed using primer software to amplify approximately 100 bp from the 3′ end of the target genes. PCR conditions were 1 min at 95 °C, followed by 40 cycles of heating at 95 °C for 15 s and 60 °C for 15 s, and 72 °C for 30 s, and final extension at 72 °C for 5 min. PCR amplification was detected by SYBR Green. The ratios of the cycle threshold (Ct) values were determined from the Bio-Rad iQ5 Optical System Software provided. To analyze the gene expression level, the ΔΔCt method was chosen and standard curves of each primer were plotted to ensure similar amplification efficiency compared with the reference gene. The *rrsA* gene, encoding the 16S RNA, and *tdh2* gene served as an endogenous control to normalize for differences in total RNA for *Z. mobilis* 8b, and *S. stipitis,* respectively.

#### Statistical analysis

Data are presented as mean ± standard error. All figures were prepared using Prism 8 (GraphPad Software, LLC).

Significant differences were statistically analyzed using IBM® SPSS® Statistics (Version 22, US). If P > 0.05 in the homogeneity of variance tests, a one-way ANOVA followed by *T* test was used. Otherwise, a nonparametric test (Kruskal–Wallis H) was used.

## Supplementary Information

**Additional file 1:** **Figure S1.** Fermentation profiles of consortia consisting of *S. stipitis* CICC1960 and* Z. mobilis *8b in 80G40XRM with “low amount” inoculums of fermentations. a Glucose assimilation profiles. b Xylose assimilation profiles. c Ethanol production profiles. Data are mean ± standard error from four replicates. **Figure S2.** Pentose metabolism and Entner-Doudoroff pathways in engineered *Z. mobilis *(1). **Table S1.** Fermentation profiles of consortia consisting of* S. stipitis *CICC1960 and *Z. mobilis *8b in 80G40XRM. **Table S2.** Fermentation profiles of* Z. mobilis* 8b, *Z. mobilis* FR1, and* Z. mobilis *FR2 in 80G40XRM. **Table S3.** Strains, plasmids, and sgRNAs used in this study. **Table S4.** Primers used in this study.

## Data Availability

Not applicable.
