# Supplementary material for: Cellulosic ethanol production by consortia of Scheffersomyces stipitis and engineered Zymomonas mobilis
Source: Biotechnol Biofuels. 2021 Nov 25;14:221. doi: 10.1186/s13068-021-02069-8 (PMC8613960; doi:10.1186/s13068-021-02069-8)
Supplement: Supplementary file 1 — Additional file 1: Figure S1. Fermentation profiles of consortia consisting of S. stipitis CICC1960 and Z. mobilis 8b in 80G40XRM with “low amount” inoculums of fermentations. a Glucose assimilation profiles. b Xylose assimilation profiles. c Ethanol production profiles. Data are mean ± standard error from four replicates. Figure S2. Pentose metabolism and Entner-Doudoroff pathways in engineered Z. mobilis (1). Table S1. Fermentation profiles of consortia consisting of S. stipitis CICC1960 and Z. mobilis 8b in 80G40XRM. Table S2. Fermentation profiles of Z. mobilis 8b, Z. mobilis FR1, and Z. mobilis FR2 in 80G40XRM. Table S3. Strains, plasmids, and sgRNAs used in this study. Table S4. Primers used in this study. [file 13068_2021_2069_MOESM1_ESM.docx]

**Supplementary Information**

Lingling Sun^1,2^, Bo Wu^1^, Zengqin Zhang^1,2^, Jing Yan^1^, Panting Liu^1,2^, Chao Song^1,2^, Samina Shabbir^1,2^, QiliZhu^1^, Shihui Yang^3^, Nan Peng^4^, Mingxiong He^1,5*^, Furong Tan^1*^

^1^ Key Laboratory of Development and Application of Rural Renewable Energy, Ministry of Agriculture and Rural Affairs, Biogas Institute of Ministry of Agriculture and Rural Affairs, Chengdu 610041, China

^2^ Graduate School of Chinese Academy of Agricultural Sciences, Beijing 100081, China

^3^ Hubei Collaborative Innovation Center for Green Transformation of Bio-resources, Environmental Microbial Technology Center of Hubei Province, Hubei Key Laboratory of Industrial Biotechnology, College of Life Sciences, Hubei University, Wuhan 430062, China

^4^ State Key Laboratory of Agricultural Microbiology, College of Life Science and Technology, Huazhong Agricultural University, Wuhan 430070, China

^5^ Chengdu National Agricultural Science and Technology Center, Chengdu 610221, China

**Correspondence:**

Mingxiong He: hemingxiong@caas.cn

Furong Tan: tanfurong@caas.cn

**
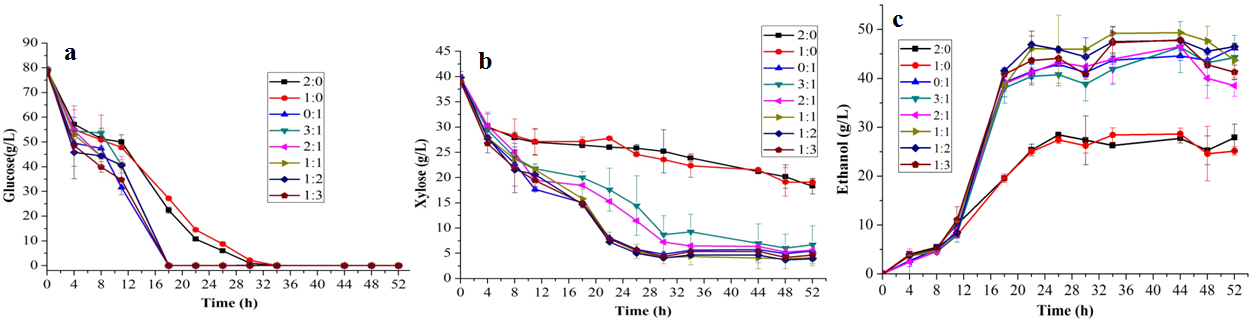
**

**Fig. S1** Fermentation profiles of consortia consisting of *S. stipitis* CICC1960 and *Z. mobilis* 8b in 80G40XRM with “low amount” inoculums of fermentations. **a** Glucose assimilation profiles. **b** Xylose assimilation profiles. **c** Ethanol production profiles. Data are mean ± standard error from four replicates

**
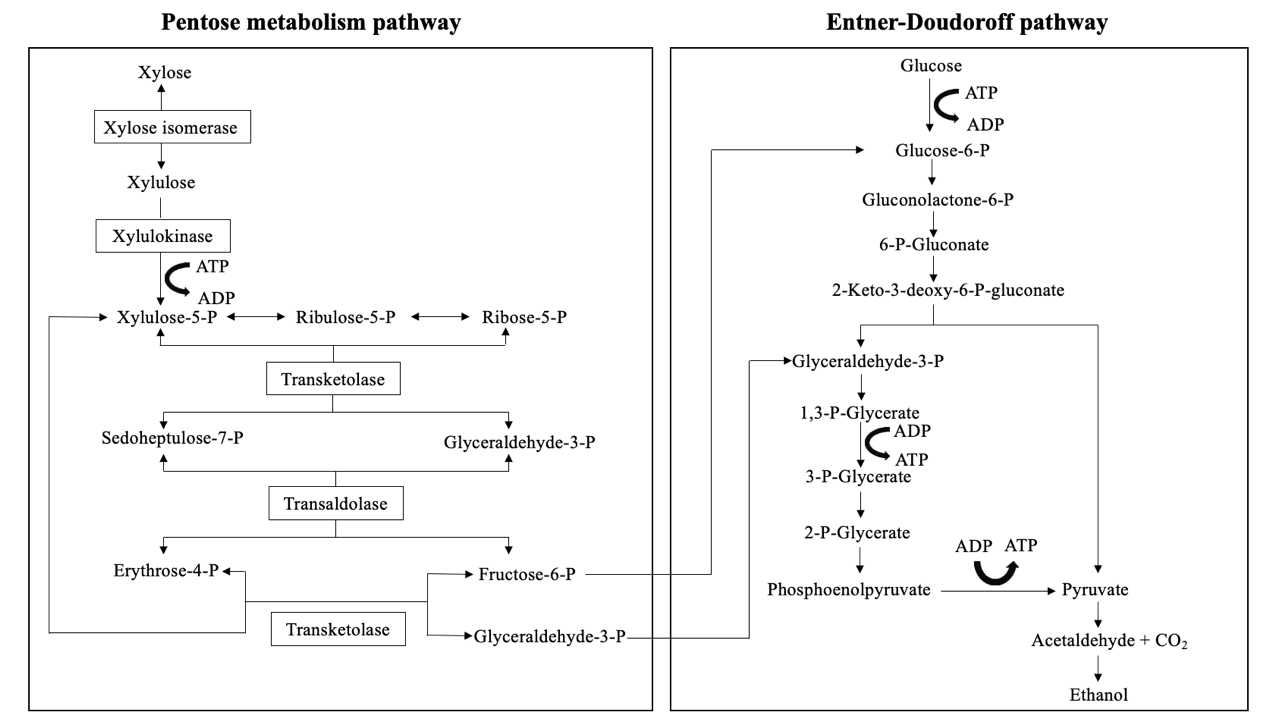
**

**Fig. S2** Pentose metabolism and Entner-Doudoroff pathways in engineered *Z. mobilis*(1)

**Table S1** Fermentation profiles of consortia consisting of *S. stipitis* CICC1960 and *Z. mobilis* 8b in 80G40XRM

| ^a^ *S. stipitis* CICC1960 : *Z. mobilis* 8b | Glucose consumption (g/L) | Xylose consumption (g/L) | Ethanol | | |
| --- | --- | --- | --- | --- | --- |
|  |  |  | Titer  (g/L) | Yield  (g/g) | Productivity (g/L/h) |
| 1:0 | 76.29 ± 2.50 | 12.71 ± 0.81 | 38.56 ± 0.29 | 0.43 ± 0.01 | 4.28 ± 0.03 |
| 0:1 | 76.29 ± 2.50 | 34.81 ± 0.55 | 53.71 ± 2.03 | 0.48 ± 0.02 | 5.10 ± 0.17 |
| 3:1 | 76.29 ± 2.50 | 21.93 ± 1.03 | 49.22 ± 0.49 | 0.50 ± 0.01 | 4.20 ± 0.08 |
| 2:1 | 76.29 ± 2.50 | 28.23 ± 0.54 | 52.70 ± 0.75 | 0.50 ± 0.00 | 4.47 ± 0.10 |
| 1:1 | 76.29 ± 2.50 | 36.60 ± 0.48^**^ | 55.76 ± 1.11 | 0.49 ± 0.01 | 4.86 ± 0.16 |
| 1:2 | 76.29 ± 2.50 | 37.06 ± 0.17^**^ | 54.84 ± 2.18 | 0.48 ± 0.02 | 5.02 ± 0.04 |
| 1:3 | 76.29 ± 2.50 | 36.73 ± 0.48^**^ | 57.21 ± 1.73 | 0.50 ± 0.01 | 4.99 ± 0.09 |

Data are mean ± standard error from four replicates. A significant difference was determined using a one-way ANOVA followed by the T-test. Asterisks indicate that this group data was significantly higher than that in *Z.mobilis* 8b mono-fermentation (none, P > 0.05; **, P < 0.01)

^a^*S. stipitis* CICC1960 : *Z. mobilis* 8b represent the initial inoculum proportion between the two species

**Table S2** Fermentation profiles of *Z. mobilis* 8b, *Z. mobilis* FR1, and *Z. mobilis* FR2 in 80G40XRM

| Strain | Glucose consumed (g/L) | Xylose consumed (g/L) | Ethanol | | |
| --- | --- | --- | --- | --- | --- |
|  |  |  | Titer (g/L) | Yield (g/g) | Productivity (g/L/h) |
| *Z. mobilis* 8b | 72.95 ± 0.73 | 31.38 ± 0.42 | 49.75 ± 0.16 | 0.48 ± 0.00 | 4.84 ± 0.01 |
| *Z. mobilis* FR1 | 72.95 ± 0.73 | 30.99 ± 0.33 | 47.94 ± 1.56 | 0.46 ± 0.01 | 4.87 ± 0.00 |
| *Z. mobilis* FR2 | 72.95 ± 0.73 | 36.20 ± 0.33** | 53.14 ± 1.06 | 0.49 ± 0.01 | 5.08 ± 0.00** |

Data are mean ± standard error from four replicates. A significant difference was determined using a one-way ANOVA followed by the T-test, if P > 0.05 in the test of homogeneity of variances. Otherwise, significant difference was determined by a nonparametric test (Kruskal-Wallis H). Asterisks indicate that the group data was significantly higher than that in *Z. mobilis* 8b fermentation (none, P > 0.05; **, P < 0.01)

**Table S3** Strains, plasmids, and sgRNAs used in this study

| Strain, plasmid or sgRNA | Characteristic | | Source |
| --- | --- | --- | --- |
| Strain |  | |  |
| *Z. mobilis* 8b | Having exogenous xylose-metabolic pathway | Kindly given by Shihui Yang, Hubei University (2) | |
| *Z. mobilis* FR1 | Derived from *Z. mobilis* 8b; ZMO0256::P*_pdc_*-*talB*-*tktA* | | This study |
| *Z. mobilis* FR2 | Derived from *Z. mobilis* FR1; ZMO0689::P*_pdc_*-*xylA*-*xylB* | | This study |
| *S. stipitis* CICC1960 |  | | China Center of Industrial Culture Collection |
| *E. coli* trans110 |  | | TransGen Biotech |
| Plasmid |  | |  |
| Pmini | addA | | Kindly given by Nan Peng, Huazhong Agricultural University (3) |
| Pmini-P*_pdc_*-*talB*-*tktA* | Derived from Pmini; carrying sgRNA (ZMO0256), ZMO0256up500, P*_pdc_*-*talB*-*tktA* and ZMO0256down500 | | This study |
| Pmini-P*_pdc_*-*xylA*-*xylB* | Derived from Pmini; carrying sgRNA (ZMO0689), ZMO0689up540, P*_pdc_*-*xylA*-*xylB* and ZMO0689down500 | | This study |

**Table S3** (continued)

| sgRNA |  |  |
| --- | --- | --- |
| sgRNA (ZMO0256) | AGATACAGAAGATTTTCTGGTACCGTTCACTGCCGCACAGGCAGCTTAGAAAGACATGGGAATGAAACAGAAAATCAGCAATTTGTTCACTGCCGCACAGGCAGCTTAGAAAGGATCCTCGAACGCGCCGAATAAG | This study |
| sgRNA (ZMO0689) | AGATACAGAAGATTTTCTGGTACCGTTCACTGCCGCACAGGCAGCTTAGAAAAATGCTGTTTCAGCAACGGATGACAACGCTTCGTTCACTGCCGCACAGGCAGCTTAGAAAGGATCCTCGAACGCGCCGAATAAG | This study |

**Table S4** Primers used in this study

| Primers | Sequence (5’ to 3’) |
| --- | --- |
| sgRNA-F | CCATTGTAGATACAGAAGATTTTCTGGTACCG |
| sgRNA-R | ACCTGAATTACTTATTCGGCGCGTTCGA |
| Pmini-F | GCCGAATAAGTAATTCAGGTTTTTTTATAAAGACCTG |
| Pmini-R | CCAGAAAATCTTCTGTATCTACAATGGCTAATTTTATTATTAGAATG |
| 130bp-F | ATTTACGATTGCTCGTCCTAAATAAATAAG |
| 130bp-R | CACTTCACTGACACCCTCATCAGTGCCAAC |
| Pmini-backbone-F | ACTAGTAGCGGCCGCTGCAG |
| Pmini-backbone-R | GTCGACACTTGCAGCTTGATATAGCCGGTA |
| ZMO0256up500-F | TATATCAAGCTGCAAGTGTCGACTTTGATGACGATTGTG |
| ZMO0256up500-R | AAGGAACGGGACACCTTGAATATATCGACCTTTTATTTTC |
| ZMO0256down500-F | GTTGATGCAAAGAGGCCCTGCCTTTACG |
| ZMO0256down500-R | CTGCAGCGGCCGCTACTAGTCTAAGAAAGTATCTTTTCCGT |
| *talB*-F | TTTGAATATATGGAGTAAGCAATGACGGACAAATTGACCTCCCT |
| *tktA*-R | CAGGGCCTCTTTGCATCAACTTACAGCAGTTCTTTTGC |
| P*_pdc_*-F | TTCAAGGTGTCCCGTTCCTT |

**Table S4** (continued)

| P*_pdc_*-R | TGCTTACTCCATATATTCAAAACACTATGTCT |
| --- | --- |
| ZMO0256-checkF | TACCACGAAAGGCGGCGTTATTCC |
| ZMO0256-checkR | GCGAAAAGCTGCGGCAAGCGATC |
| ZMO0689up540-F | GCTATATCAAGCTGCAAGTGTCGACGTCTTTGCGTCCAGAAAAGACAGCA |
| ZMO0689up540-R | GAAAAAGGAACGGGACACCTTGAAAATCCTTGTTTCTTTCTTAACTAACC |
| ZMO0689down500-F | TATCCGTGCTTTCAAAGCCGGCAAGCATGT |
| ZMO0689down500-R | CTGCAGCGGCCGCTACTAGTGCACCGAGAAACGTGAA |
| *xylA*-F | TTTGAATATATGGAGTAAGCAATGCAAGCCTATTTTGACCAGCTCGA |
| *xylB*-R | CCGGCTTTGAAAGCACGGATATTACGCCATTAATGGCAGAA |
| ZMO0689-checkF | ATCAATGGATCTCCGAAGAGGCTT |
| ZMO0689-checkR | CATCATCGACTGGTTAGCATGGC |
| ZMO0366-F | GACGTCATTCCGAGGCTAGTAA |
| ZMO0366-R | AAGGCAGCAACGGATACACC |
| HGT1-F | ATGTGTGCTTCGTGGATGGT |
| HGT1-R | GTGCTATTCCGCCCACAAAA |
| HGT2-F | CCAGGCTCAGACATCCAAGG |
| HGT2-R | GCACCGACCATCCAGAAGAA |
| MIG1-F | AGAACTCACACCGGCGAAAA |
| MIG1-R | CCAGTGATCTGACCGTTGCT |
| MIG2-F | ATGCTGTTGCCGTCTCGTAA |
| MIG2-R | ATCCTCGCTGGAATTGGTGG |
| QUP2-F | GGGATCTGTTCCGTGCTCTC |
| QUP2-R | CATGGCTTGAGCTGCCTTTC |
| RGT2-F | GGTCCTCTTTGTTGGGCTGT |
| RGT2-R | GGCAATACCCCAGTTCCACA |
| SNF3-F2 | GTTGGTAGAGCGATCTCGGG |
| SNF3-R2 | AAACGACGGAACCTCTGACC |
| ZMO0293-F | GACGTCATTCCGAGGCTAGCAA |
| ZMO0293-R | AAGGCAGCAACGGATACCCC |
| XUT4-F | GCCAGGAGAGCAAGTGTCAT |
| XUT4-R | ATGGTGGTACTGGCGAAAGG |
| XUT5-F | GCAATCAGCAGCCCAAAACA |
| XUT5-R | TGTCTGGAGGGGCTAGTTCA |
| XUT7-F | TTTCTGGGGTGGGTGTAGGA |
| XUT7-R | AGGCTTCTCCCTTGCATCAC |
| 16s-F | TCAACTATAGACCAGTAAGT |
| 16s-R | AGAACATAGAAGAGGTAAGT |
| tdh2-F | AAGGCTTGATGACTACTGTTCA |
| tdh2-R | CTTCATAGCAGCACAGATTTCC |

**References**

1. Zhang M, Eddy C, Deanda K, Finkelstein M, Picataggio S. Metabolic engineering of a pentose metabolism pathway in ethanologenic *Zymomonas mobilis*. Science. 1995;267(5195):240-3.

2. Yang S, Vera JM, Grass J, Savvakis G, Moskvin OV, Yang Y, et al. Complete genome sequence and the expression pattern of plasmids of the model ethanologen *Zymomonas mobilis* ZM4 and its xylose-utilizing derivatives 8b and 2032. Biotechnol Biofuels. 2018;11:125.

3. Wang X, Wu B, Sui X, Zhang Z, Liu T, Li Y, et al. CRISPR-mediated host genomic DNA damage is efficiently repaired through microhomology-mediated end joining in *Zymomonas mobilis*. J Genet Genomics. 2021.
